# Supplementary material for: Bayesian refinement of protein structures and ensembles against SAXS data using molecular dynamics
Source: PLoS Comput Biol. 2017 Oct 18;13(10):e1005800. doi: 10.1371/journal.pcbi.1005800 (PMC5662244; doi:10.1371/journal.pcbi.1005800)
Supplement: S1 Archive — (BZ2) [file pcbi.1005800.s011.tar.bz2 › gromacs_4.62_bayesian_saxs_refi/share/html/online/water.html]

Getting started - Water


| Getting started - Water Main Table of Contents |

**VERSION 4.6  
Sat 19 Jan 2013**

---

## Water

Now you are going to simulate 216 molecules of SPC water
(Berendsen et al., 1981)
in a cubic box. In this example the GROMACS
software team already generated most of the neccesary input
files. The files needed in this example are:

- Initial structure of a box of 216 water molecules (.gro)
- Topology file of water (.top)
- Molecular Dynamics parameter file (.mdp)

Change your directory to tutor/water :
  
  

|  |  |  |
| --- | --- | --- |
|  | cd tutor/water  \| |

  
Let's first have a look at the coordinate file:
  
  

|  |  |  |
| --- | --- | --- |
|  | more conf.gro   \| |

  
Or to view the water box graphically:
  
  

|  |  |  |
| --- | --- | --- |
|  | rasmol spc216.pdb   \| |

  
Have a look at the topology file:
  
  

|  |  |  |
| --- | --- | --- |
|  | more topol.top   \| |

  
Have a look at the MD-parameters file:
  
  

|  |  |  |
| --- | --- | --- |
|  | more grompp.mdp   \| |

  
Since all the neccesary files are available, we are going to
preprocess the input files to create a run input
(.tpr) file.
This run input file is the only input file for the
MD-program mdrun.
  
  

|  |  |  |
| --- | --- | --- |
|  | grompp -v   \| |

  
The run input file is only viewable with the program
gmxdump.
In this way it is possible to check if the preprocessor
grompp worked well.
The output may seem rather cryptic, so don't panic when you don't
understand every single item in it.
  
  

|  |  |  |
| --- | --- | --- |
|  | gmxdump -s topol.tpr \| more   \| |

  
Now it's time to start the simulation of 20 picoseconds.
  
  

|  |  |  |
| --- | --- | --- |
|  | mdrun -v   \| |

  
After the MD simulation is finished, it is possible to view the
trajectory with the ngmx program:
  
  

|  |  |  |
| --- | --- | --- |
|  | ngmx   \| |

  

When the program starts, you must select a group of atoms to view. In
our case that will be "SOL" (for solvent) or "System", which is the
same for a box of water as we have. Select one and click OK. Then
select Display->Animate from the menu. Use the buttons to see your
water moving (note: "Play" steps one frame forward; "Fast Forward"
plays; "Rewind" skips back to the beginning of the trajectory).

---

## Analysis of the simulation

1. Calculate a radial distribution function of the Oxygen atoms. The
   index file index.ndx
   contains one group with all the oxygen atoms.
     
     

   |  |  |  |
   | --- | --- | --- |
   |  | g\_rdf -n index   \| |

     
   The program will ask you for how many groups you want the calculate
   the RDF, answer 1. Now,
   view the output graph of g\_rdf.
   The file is already prepared to produce a nice graph in the
   xmgrace program.
     
     

   |  |  |  |
   | --- | --- | --- |
   |  | xmgrace rdf.xvg   \| |

     
   Which shows you the radial distribution function for Oxygen-Oxygen in
   SPC water. The first peak is at roughly 0.28 nm, and has a height of 2.8.
   This means that the relative density of oxygen atoms in water,
   is almost 3 times higher at a distance of 0.28 nm from another oxygen atom.
   Obviously this is due to strong hydrogen bonding in liquid water.
2. We can also do a direct analysis of the number of hydrogen bonds in
   liquid water, based on O-O distance and O-H ... O angle.
     
     

   |  |  |  |
   | --- | --- | --- |
   |  | g\_hbond   \| |

     
   Select twice 0, when asked. Check the output with
     
     

   |  |  |  |
   | --- | --- | --- |
   |  | xmgrace hbnum.xvg   \| |

     
   What is the number of hydrogen bonds per water
   molecule? What is the maximum possible number?
3. As a further test of the simulation we will compute the self diffusion constant
   of water. This can be computed from the **Mean square displacement** (MSD)
   function. The MSD determines how much on average a molecule has moved
   with time. After a certain time period there is no correlation between the
   starting position and the current position and the square displacement will
   be linear with time. The slope of the MSD function is the linearly proportional
   to the self diffusion constant.
     
     

   |  |  |  |
   | --- | --- | --- |
   |  | g\_msd -n index   \| |

     
   View the output
     
     

   |  |  |  |
   | --- | --- | --- |
   |  | xmgrace msd.xvg   \| |

     
   Check that the graph is roughly linear. The g\_msd program also
   computes the diffusion constant D for you. The experimental value
   is roughly 2.3 x 10^-5^ cm^2^/s. The simulation
   result is clearly too high, due to the simplicity of the model.

---


### Go to the next step


---
